# Proliferative endocrine effects of adipose tissue from obese animals on MCF7 cells are ameliorated by resveratrol supplementation

**DOI:** 10.1371/journal.pone.0183897

**Published:** 2017-09-05

**Authors:** Christopher F. Theriau, O’Llenecia S. Sauvé, Marie-Soleil Beaudoin, David C. Wright, Michael K. Connor

**Affiliations:** 1 School of Kinesiology and Health Science, Muscle Health Research Centre, York University, Toronto, ON, Canada; 2 Department of Human Health and Nutritional Sciences, University of Guelph, Guelph, ON, Canada; University of PECS Medical School, HUNGARY

## Abstract

Obesity is clearly associated with an increased risk of breast cancer in postmenopausal women. The purpose was to determine if obesity alters the adipocyte adipokine secretion profile, thereby altering the adipose-dependent paracrine/endocrine growth microenvironment surrounding breast cancer cells (MCF7). Additionally, we determined whether resveratrol (RSV) supplementation can counteract any obesity-dependent effects on breast cancer tumor growth microenvironment. Obese ZDF rats received standard chow diet or diet supplemented with 200 mg/kg body weight RSV. Chow-fed Zucker rats served as lean controls. After 6 weeks, conditioned media (CM) prepared from inguinal subcutaneous adipose tissue (scAT) was added to MCF7 cells for 24 hrs. Experiments were also conducted using purified isolated adipocytes to determine whether any endocrine effects could be attributed specifically to the adipocyte component of adipose tissue. scAT from ZDF rats promoted cell cycle entry in MCF7 cells which was counteracted by RSV supplementation. RSV-CM had a higher ratio of ADIPO:LEP compared to ZDF-CM. This altered composition of the CM led to increased levels of pAMPK^T172^, p27, p27^T198^ and AdipoR1 while decreasing pAkt^T308^ in MCF7 cells grown in RSV-CM compared to ZDF-CM. RSV-CM increased number of cells in G0/G1 and decreased cells in S-phase compared to ZDF-CM. Co-culture experiments revealed that these obesity-dependent effects were driven by the adipocyte component of the adipose tissue. Obesity decreased the ratio of adiponectin:leptin secreted by adipocytes, altering the adipose-dependent growth microenvironment resulting in increased breast cancer cell proliferation. Supplementation with RSV reversed these adipose-dependent effects suggesting a potential for RSV as a nutritional supplementation to improve breast cancer treatment in obese patients.

## Introduction

Breast cancer is a dynamic, multi-factorial and inherently complex disease. Despite this, the tumor growth environment within each individual patient is much more stable and uniform, since the majority of factors within this environment are originate from predictable determinants of patient physiology. Thus, targeting this growth microenvironment therapeutically may elicit more predictive treatment outcomes across patients and over a broader range of tumor types. Since the vast majority of tumors are surrounded by adipocytes and adipocytes serve as an active endocrine tissue, there may exist direct effects of adipose on tumor growth [1,2] making adipocytes, and adipose as a whole, viable targets for novel cancer therapeutic strategies. Relevant to this, an obesity/breast cancer link has existed for almost 50 years with increased adiposity being associated with an increased risk of breast cancer development [3]. Also, obese postmenopausal women are 50% more likely to develop breast cancer compared to their lean counterparts [4,5]. Furthermore, obese women are more likely to suffer from metastatic breast cancer and have a poorer clinical outcome than non-obese women [4]. Taken together, there is a clear connection between adiposity and breast cancer emphasizing the existence of a role of adipose tissue in regulating cancer progression.

Traditionally, adipocytes have been thought to be an inert storage depot, but in fact adipose tissue secretes over 400 different adipokines into the extracellular space and the systemic circulation, making it an important contributor to the endocrine/paracrine local environments that exist throughout the body [6]. Specifically, adiponectin (ADIPO) and leptin (LEP) have been shown to elicit growth effects on tumor cells and their levels are altered as adiposity changes [7–9]. ADIPO levels are inversely proportional to adiposity and it induces cell cycle exit in MCF7 cells via AMPK mediated phosphorylation of p27 at T198 resulting in increased p27 protein stability and cell cycle exit [7,10,11]. LEP secretion is directly proportional to adiposity and it elicits the opposite cell cycle effects to those of ADIPO by activating Akt and promoting cytoplasmic localization of p27 [8,12]. The lower levels of ADIPO and higher levels of LEP in obese individuals correlate with a greater incidence of tumor formation [2]. Furthermore, serum ADIPO is reduced while LEP is increased breast cancer patients compared to healthy women [13,14]. Since ADIPO and LEP activate antagonistic intracellular signaling pathways [15], it appears that the ratio of ADIPO:LEP may be a more reliable predictor of cancer incidence and outcome in breast cancer patients [2,16]. Visceral adipose tissue of “obese” high fat diet (HFD) fed animals has been shown to promote breast cancer cell cycle entry by decreasing pAMPK^T172^, p27, p27^T198^ and AdipoR1 protein levels while increasing pAkt^T308^ [15]. Conversely, adipose from “lean” animals elicited the opposite response [15]. The higher ADIPO:LEP ratio secreted by lean adipose compared to obese adipose tissue seems to underlie these effects. Thus, the tumor growth microenvironment produced by the adipokine secretion profile of adipose tissue of obese patients likely plays a direct role in controlling breast cancer growth.

The search for novel and effective cancer chemo-preventative substances has expanded to include the study of various naturally occurring compounds. Resveratrol (RSV) is a phytoalexin produced by plants and is concentrated in the skin of red grapes. RSV elicits established effects on metabolism, but these are far from completely characterized. High fat diet-fed rodents supplemented with RSV display an altered adipokine profile compared to those without supplementation, with ADIPO increasing and LEP decreasing and these effects appear to be mediated by AMPK activation within the adipocytes [17–20].

The current study examined the effects of RSV supplementation on adipokine secretion in white adipose tissue from ZDF rats. We hypothesized that adipose from obese ZDF rats will create a growth promoting tumor growth microenvironment for MCF7 cells and that this effect will be specifically due to the adipocyte component of the adipose tissue. Furthermore, RSV supplementation will counteract these effects by altering the adipokine secretion profile of the obese adipose tissue. This would highlight RSV as a potential adjuvant therapy for obese breast cancer patients.

## Materials and methods

### Animals

All protocols followed Canadian Council on Animal Care guidelines and were approved by the University of Guelph and York University Animal Care Committees. For co-culture experiments, five 10 week old ZDF and five age matched lean Zucker rats (Charles River, St. Constant, QC, Canada) were singly housed in standard clear, plastic cages. Male rats were used to remove any effects of estrogen on the adipose tissue adipokine secretion profile as previously described [15]. Animals habituated in a temperature and humidity (50–60%) controlled room for seven days and were fed a standard show diet (Purina 5012 Chows, Ralston Purina, St. Louis, MO) and water *ad libitum*.

For RSV supplementation experiments, four-week old male ZDF rats were housed as previously described [21]. Following acclimatization, animals were fed either a standard powdered chow diet (Purina 5008 diet; Purina; ZDF) or a diet supplemented with ~200 mg/kg body weight RSV (Cayman Chemical, Ann Arbor, MI, USA; ZDF+RSV) for 6 weeks (4). While this dose of RSV is likely not attainable through diet alone, it is similar to many other rodent-based reports in the literature [20,22,23]. Zucker rats served as lean controls.

### Adipocyte isolation, co-culture and conditioned media

Since we have previously shown that “obese” adipose tissue can induce cell cycle entry in MCF7 cells [15], initial experiments were designed to evaluate whether the adipose-dependent effects on MCF7 cell cycle regulation were specifically due to the adipocytes. By eliminating the inflammatory and stromal cells we are able to infer the paracrine effects of adipocytes alone in regulating MCF7 cell signaling and proliferation. Adipose tissue from the inguinal subcutaneous (scAT) depot was harvested and purified adipocytes were isolated as previously described [24,25]. Briefly, adipose tissue was minced into very fine pieces, until a paste-like consistency was established, and treated with type II collagenase (0.4 mg/ml) for 10 minutes at 37°C in a shaking water bath. Following collagenase treatment, adipocytes were passed through a coarse metal sieve to remove large, undigested and unseparated fat pieces. The filtered adipocytes were washed with improved modified Eagle’s medium without phenol red (IMEM, Wisent, Montreal, PQ, CAN) supplemented with 5% fetal bovine serum (FBS, Hyclone, Thermo Fisher Scientific, Whitby, ON) and centrifuged for 20 seconds at 1000 x g. Following centrifugation, the adipocytes float on top of the wash media while immune cells, stromal cells and blood cells pellet at the bottom of the tube, which allows for separation from the adipocytes. The wash media was then aspirated to remove non-adipocyte particulate matter from the preparation. Adipocytes were then washed 3 more times and allowed to equilibrate in IMEM/5% charcoal stripped FBS (cFBS, Wisent) for 45 mins. This adipocyte isolation procedure results in a “clean” preparation, as evidenced by the lack of contamination by other cell types when viewed under the microscope. Following isolation, adipocytes were counted according to published protocols (20, 21). Average adipocyte cell volume was calculated and a measure of the concentration of adipocytes (number of cells/ml) in our preparation was determined. Pre-determined adipocyte volumes were added to 6-well plates containing MCF7 cells for 24 hours. For all experiments MCF7 cells were treated at 80% confluence to ensure a uniformity of MCF7:adipocytes between lean and obese experiments. Thus, each experiment yielded comparable total adipocyte volumes to directly compare the effects of obesity per adipocyte volume. In these co-culture experiments the adipocytes float on the top of the culture media (2 ml total volume) due to their hydrophobic nature. This results in complete separation of the adipocytes from the MCF7 cells which adhere to the plate. After 24 hours the media was collected for adipokine measurements and cells were subsequently washed three times with warm PBS which removes all adipocyte components and ensured MCF7 protein extracts were free of any adipocyte-derived proteins. By utilizing this mode of adipose preparation and co-culture design we would be able to isolate the contribution of the adipocytes alone on MCF7 proliferation and compare to those effects to experiments using conditioned media (CM) prepared from crude adipose tissue (see below). While it is not possible to create the exact adipocyte:cancer cell ratio that would occur *in vivo*, we can examine direct paracrine effects of adipocytes on MCF7 proliferation.

Subsequent experiments were conducted using conditioned media (CM) prepared from crude whole adipose preparations, as this model has been successfully employed to examine the effects of adipose on breast cancer growth (30). All CM was prepared from inguinal adipose depots as previously described [15,21]. Briefly, the epididymal fat was weighed, minced into ~5–10 mg pieces and immediately placed in 50 ml vented conical tubes containing Alpha Modified Eagle’s Medium (AMEM; 7.5 ml/g tissue; Wisent) supplemented with 10% fetal bovine serum (Hyclone,), 2% antimicotic/antibiotic (Wisent), 1 mM sodium pyruvate (Sigma, Oakville, ON), non-essential amino acids (Sigma), and 10 μg/ml insulin from human pancreas (Wisent) under sterile conditions and incubated for 24 hours at 37°C with 5% CO_2_. After 24 hours, conditioned media (CM) was then collected and stored at -84°C for future use. We have conducted numerous experiments using this type of model to establish the efficacy of combining rat adipose tissue and human epithelial cells [15]. In order to ensure that our mass:volume preparation of CM (1 g:7.5 ml) was not skewed by the presence of vast differences in adipose cellular content, we diluted 10–15 mg sections of adipose 30:1 in RIPA buffer for protein extraction. Equal volumes of lysate (25 μl) were subjected to SDS-PAGE using 12% gels and membranes were probed for total Akt and β-actin. This demonstrated an equivalency of specific protein content between groups in the preparation of CM [15].

### Conditioned media adipokine measurements

The levels of ADIPO and LEP secreted into the media by isolated purified adipocytes and crude adipose(CM) preparations were determined using rat adiponectin (BioVision, Milpitas, CA) and leptin (R&D Systems, Minneapolis, MN) ELISA kits as per manufacturer instructions, respectively. The levels of each adipokine were expressed in ng/ml and nM values.

### Cell culture

MCF7 cells (ATCC, Manassas, VA) were maintained in AMEM supplemented with 10% FBS, 2% anti-micotic/anti-biotic, 1% 100 mM sodium pyruvate, 1% non-essential amino acids, and 10 μg/ml insulin from human pancreas at 37°C and 5% CO_2_. Initial purified adipocyte co-culture experiments were designed to determine whether obese ZDF adipocytes were capable of inducing cell cycle entry in arrested G0/G1 synchronized MCF7 cells. To induce G0/G1 arrest, MCF7 cells were cultured in 6-well plates in IMEM supplemented with 5% cFBS, 2% anti-micotic/anti-biotic for 24hrs until arrested and subsequently exposed to increasing scAT adipocyte volumes. In order to determine the effect of increasing the ADIPO:LEP ratio within the adipocyte/MCF7 cell co-cultures, 18 nM human globular adiponectin (gADIPO; Peprotech, Rocky Hill, NJ) was added to wells containing the highest adipocyte content. The 18 nM concentration was used based on previous titration experiments in our lab and is within the linear response range of gADIPO that elicits alterations in MCF7 proliferation.

After establishing that obese adipocytes can induce cell cycle arrest in quiescent MCF7 cells, we then wished to examine whether RSV supplementation of ZDF rats could alter adipokine secretion profile of whole adipose tissue in a way that it would induce cell cycle withdrawal in proliferating MCF7 cells. To do this we prepared conditioned media (CM) using adipose from the various treatment groups. MCF7 cells were plated in Media 199 (Sigma-Aldrich, Oakville, ON, Canada) supplemented with 10% FBS and 2% anti-micotic/anti-biotic and treated with either ZDF-CM, ZDF+RSV-CM or lean Zucker-CM for 24hrs. MCF7 cells grown in non-conditioned Media 199 (10% FBS and 2% anti-micotic/anti-biotic) served as untreated controls (UT).

### Immunoblotting

The levels of specific proteins across treatments were measured using standard SDS-PAGE protocols. Following protein transfer, polyvinylidene fluoride (PVDF) membranes (Bio-rad, Mississauga, ON, CAN) were incubated with primary antibodies: p27^Kip1^ (BD Biosciences); p27^T198^ (R&D Systems, Minneapolis, MN); cyclin E, phospho-Akt^T308^, Akt, phospho-AMPK^T172^ and AMPK (Cell Signaling, Pickering, ON,CAN); AdipoR1 (Santa Cruz Biotech, Santa Cruz, CA) and β-actin (Abcam, Cambridge, MA). Horseradish peroxidase-conjugated secondary antibodies (Santa Cruz) allowed for the determination of protein levels using Immobilon enhanced chemiluminescence substrate (Millipore, Whitby, ON, CAN), detected on a Kodak *In Vivo* Pro imaging system (Marketlink Scientific, Burlington, ON, CAN) and quantified using Carestream Software (Carestream Health, Rochester, NY, USA).

### Cell cycle analyses

Treated MCF7 cells were fixed by drop wise addition of ice-cold 70% ethanol, washed in PBS and re-suspended in a propidium iodide/RNAse solution prior to FACS analyses (FACSCalibur, BD Biosciences, Mississauga, Canada). Cell cycle profiles were determined using Mod-fit software (Verity Software House, Topsham, ME).

### Statistical analyses

All values are expressed as means ± SEM of (n = 3–6, as indicated) and statistical analyses were performed using a one-way ANOVA with Tukey’s multiple comparisons test used when main effects were found, with significance levels being set at p<0.05. For the isolated adipocyte experiments, 3–4 animals were used/group while 5–6 animals/group were used for CM experiments.

## Results

### Obese adipocytes promote cell cycle entry in arrested MCF7 cells

**Fig 1:**
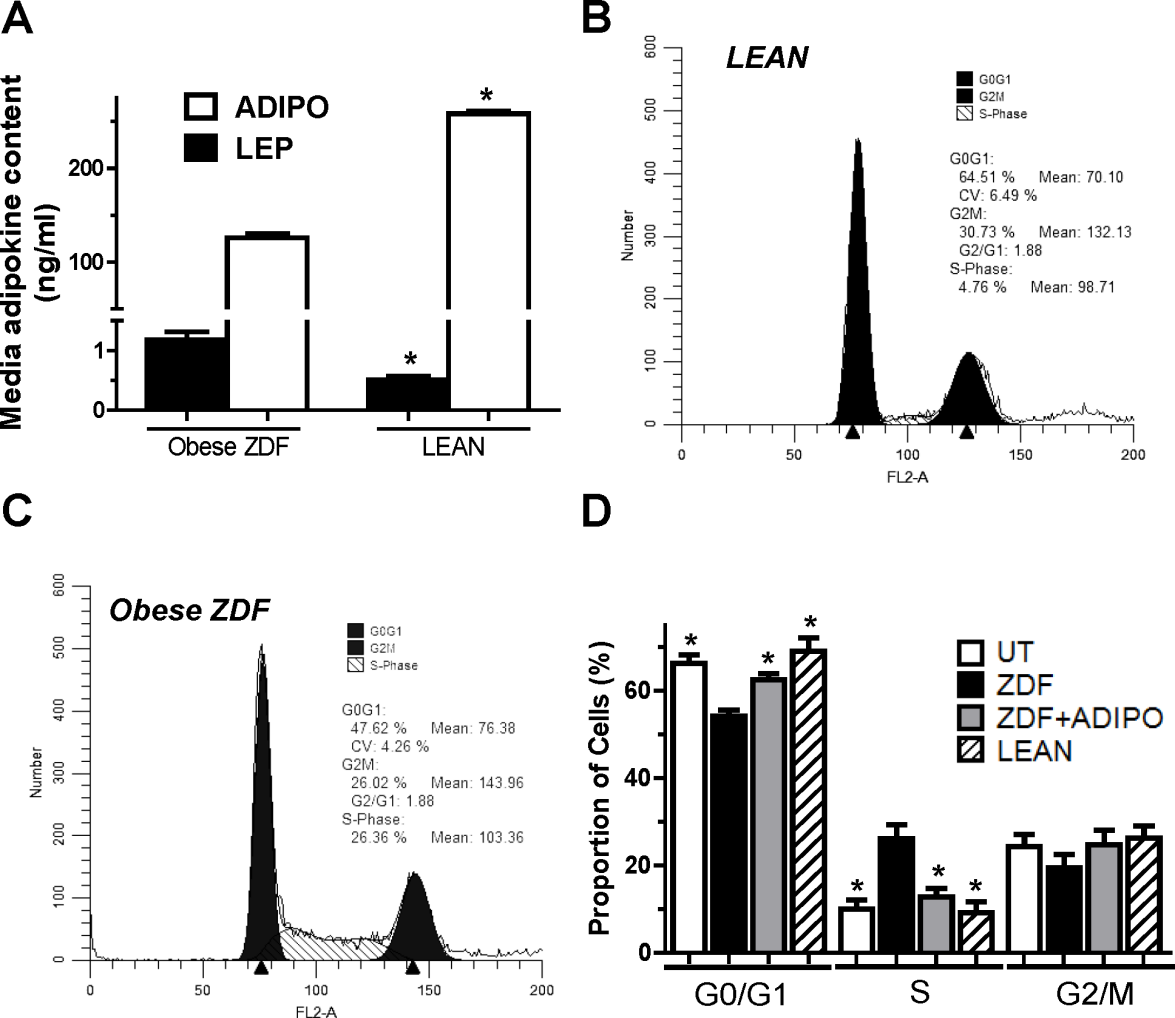
Co-cultured subcutaneous adipocytes elicit cell cycle changes in MCF7 cells. A: Graphical representation of ADIPO and LEP concentration (ng/ml) in media co-cultured with isolated subcutaneous adipocytes and MCF7 cells. B-C: Typical cell cycle profiles in MCF7 cells treated with lean Zucker adipocytes (4.4×10^6^) and ZDF adipocytes (1.6×10^5^). D: Graphical representation of multiple cell cycle profile experiments observing effects of adipocyte co-culture with ZDF (closed bars), ZDF+ 9 nM ADIPO (grey bars) and lean Zucker (hatched bars) in arrested MCF7 cells. One-way ANOVAs showed significant main effects for all experiments (p<0.05). * denotes significantly different from ZDF (p<0.05) as determined by Tukey’s post-hoc tests (n = 5/group).

Media from adipocyte/MCF7 cell co-culture experiments contained altered ADIPO and LEP levels depending on the origin of the adipocytes used. “Lean” animals weighed 342±5.2 g (n = 5) while “obese” ZDF animals were significantly heavier (p<0.05), weighing 542±22.6 g (n = 5). Media from “lean” adipocyte co-cultures contained 258.2±2.5 and 0.51±0.06 ng/ml of ADIPO and LEP, respectively (Fig 1A). This resulted in a stoichiometric ADIPO:LEP ratio of 509:1 in the growth microenvironment (media) created by the “lean” adipocytes. Conversely, the media from co-culture experiments using “obese” ZDF adipocytes contained 125.7±4.3 and 1.18±0.13 ng/ml of ADIPO and LEP, respectively (Fig 1A). This resulted in an 80% reduction (p<0.05) in the ADIPO:LEP ratio to 106:1 in the culture media created by the “obese” adipocytes compared to that seen in “lean” co-culture media.

To determine whether this altered ADIPO and LEP secretion from “obese” ZDF adipocytes elicited any growth effects on the MCF7 cells in co-culture experiments, we measured cell cycle profiles using FACS analyses (Fig 1B and 1C). When incubated with adipocytes from scAT of ZDF rats, 54.1±2.7% of MCF7 cells were in G1, which represented a 22% and an 18% reduction (p<0.05) compared to cells co-cultured with adipocytes from “lean” animals and UT arrested MCF7 cells, respectively (Fig 1D). Concomitantly, 26.0±6.5% of MCF7 cells were in S-phase when co-cultured with “obese” ZDF adipocytes, representing a 2.8-fold increase above MCF7 cells cultured with “lean” adipocytes and a 2.6-fold increase compared to UT arrested MCF7 cells (p<0.05). This suggests that “obese” adipocytes create a growth environment that induces MCF7 cell cycle entry while “lean” adipocytes continue to promote cell cycle arrest in these same cells. To examine the effect of altering the ADIPO:LEP ratio alone on tumor growth environment, we increased this ratio by adding 18 nM gADIPO to the “obese” ZDF co-cultures. The addition of ADIPO caused a reduction in the number of cells in S-phase and an increase in the number of cells in G1 (p<0.05), almost completely removing the proliferative effects of the “obese” adipocytes (Fig 1D).

### Co-culture with purified “lean” and “obese” subcutaneous adipocytes differentially affects intracellular signaling and cell cycle proteins in MCF7 cells

**Fig 2:**
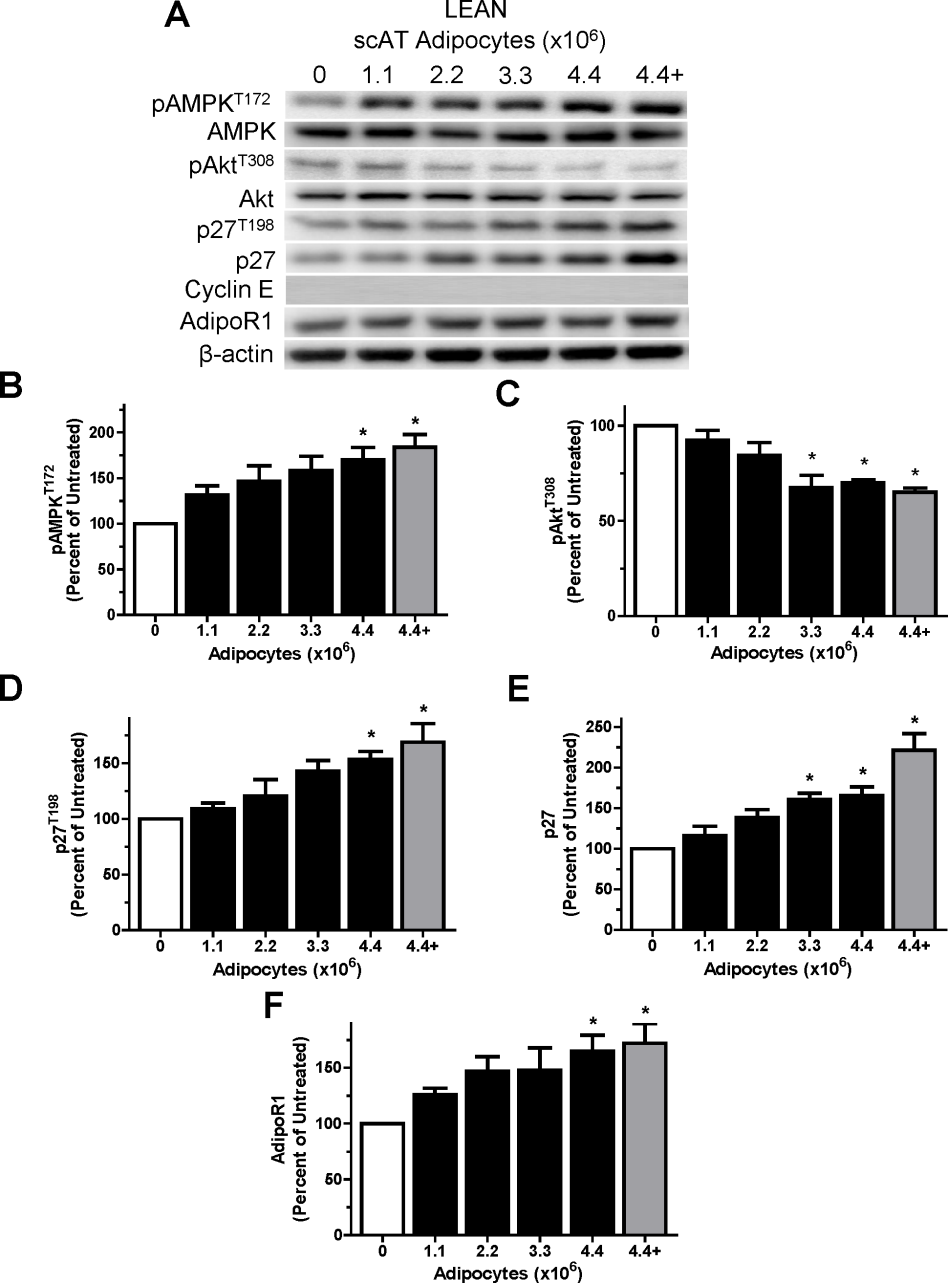
Lean Zucker subcutaneous adipocytes co-culture positively affects MCF7 cell cycle proteins in does-dependent manner. A: Representative western blots for selected proteins showing the effects of treatment with UT (open bar) or lean Zucker scAT increasing adipocyte number (closed bars) or the highest adipocyte number (4.4×10^6^) plus ADIPO (18 nM; grey bar) co-culture treatment for 24 hours in arrested MCF7 cells. B-F: Graphical representations of multiple experiments showing the effects of lean Zucker scAT increasing adipocyte number and the addition of ADIPO (9 nM) on pAMPK^T172^, pAKT^T308^, p27, p27^T198^ and AdipoR1 protein levels. β-actin was used as a loading control. One-way ANOVAs showed significant main effects for all experiments (p<0.05). * denotes significantly different from ZDF (p<0.05) as determined by Tukey’s post-hoc tests (n = 5/group).

Following 24 hour co-culture with “lean” or “obese” scAT adipocytes, MCF7 cells were harvested and the levels of signaling and cell cycle proteins were measured. Purified “lean” subcutaneous adipocytes increased pAMPK^T172^, p27, p27^T198^ and AdipoR1 protein levels (p<0.05) in the MCF7 cells while decreasing pAkt^T308^ protein levels (p<0.05) in a dose-dependent manner (Fig 2A–2F). The level of the cell cycle protein cyclin E was undetectable in all MCF7 “lean” subcutaneous adipocyte co-cultures. Addition of gADIPO to the co-cultures with the highest adipocyte numbers (4.4×10^6^) caused no further increases (p>0.05) in all measured proteins (Fig 2A–2F). No changes in total AMPK and Akt were evident across treatment groups (p>0.05).

**Fig 3:**
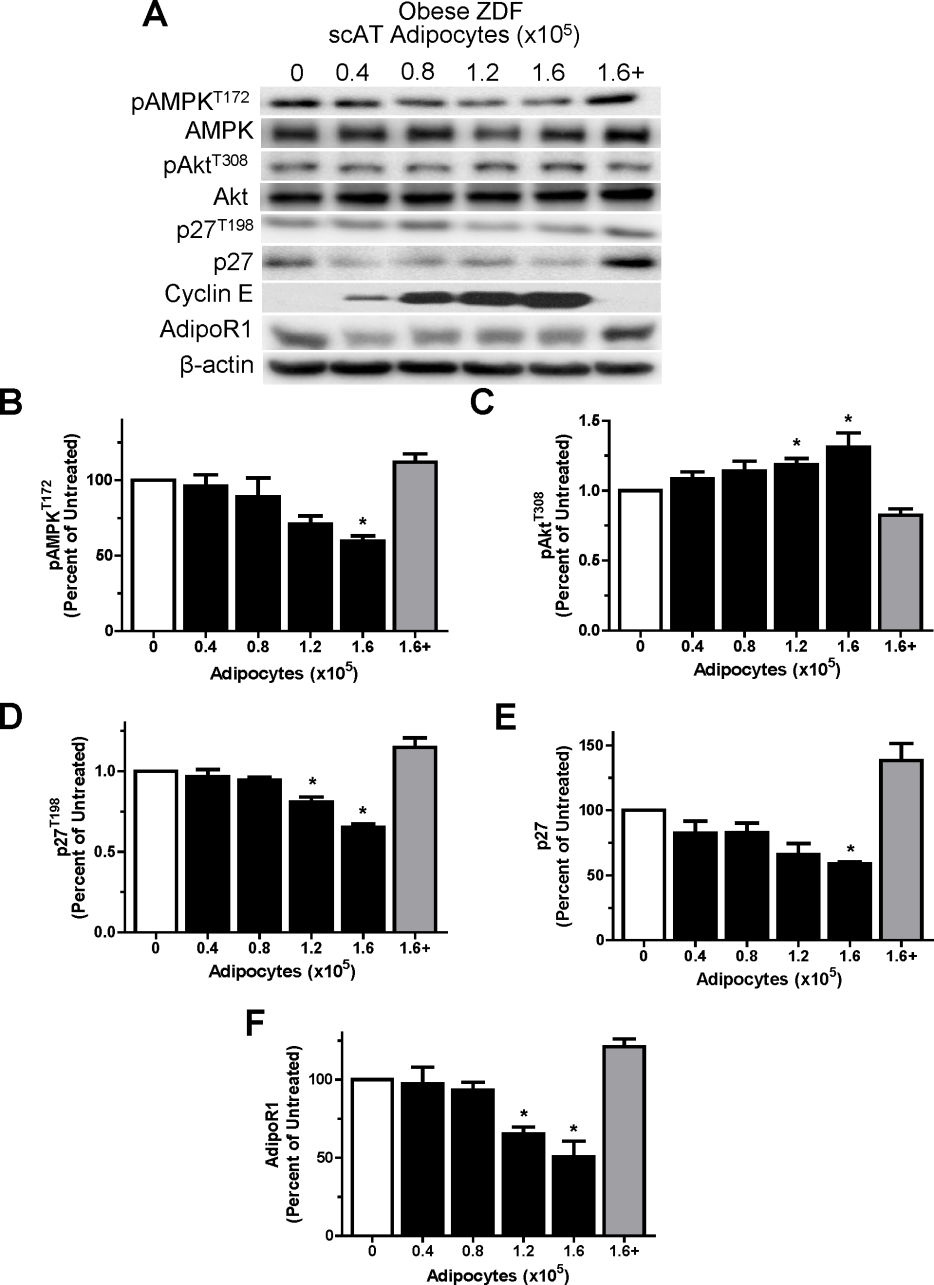
ZDF subcutaneous adipocytes co-culture negatively affects MCF7 cell cycle proteins in does-dependent manner. A: Representative western blots for selected proteins showing the effects of treatment with UT (open bar) or ZDF scAT increasing adipocyte number (closed bars) or the highest adipocyte number (1.6×10^5^) plus ADIPO (18 nM; grey bar) co-culture treatment for 24 hours in arrested MCF7 cells. B-F: Graphical representations of multiple experiments showing the effects of ZDF scAT increasing adipocyte number and the addition of ADIPO (18 nM) on pAMPK^T172^, pAKT^T308^, p27, p27^T198^ and AdipoR1 protein levels. β-actin was used as a loading control. One-way ANOVAs showed significant main effects for all experiments (p<0.05). * denotes significantly different from ZDF (p<0.05) as determined by Tukey’s post-hoc tests (n = 5/group).

In contrast, purified subcutaneous adipocytes from “obese” ZDF animals activated intracellular signaling pathways and responses that suggested the induction of cell cycle entry within the MCF7 cells. Co-culture with “obese” subcutaneous adipocytes elicited dose–dependent decreases in pAMPK^T172^, p27, p27^T198^ and AdipoR1 protein levels (p<0.05), while markedly increasing pAkt^T308^ and cyclin E protein levels (p<0.05) in MCF7 cells (Fig 3A–3F). Addition of 18 nM gADIPO to the co-cultures with the highest adipocyte numbers (1.6x10^5^) resulted in a “rescuing” of the MCF7 cells with protein expression being very similar to that in untreated MCF7 cells, effectively abolishing the effect of the “obese” adipocytes (Fig 3A–3F last lane vs. first lane). Interestingly, the level of p27 climbed to levels that were significantly higher (p<0.05) than those in UT cells (Fig 3A & 3E). No changes in total AMPK and Akt were evident across treatment conditions.

### RSV supplementation alters the adipokine secretion profile of scAT which affects MCF7 cell cycle profiles

**Fig 4:**
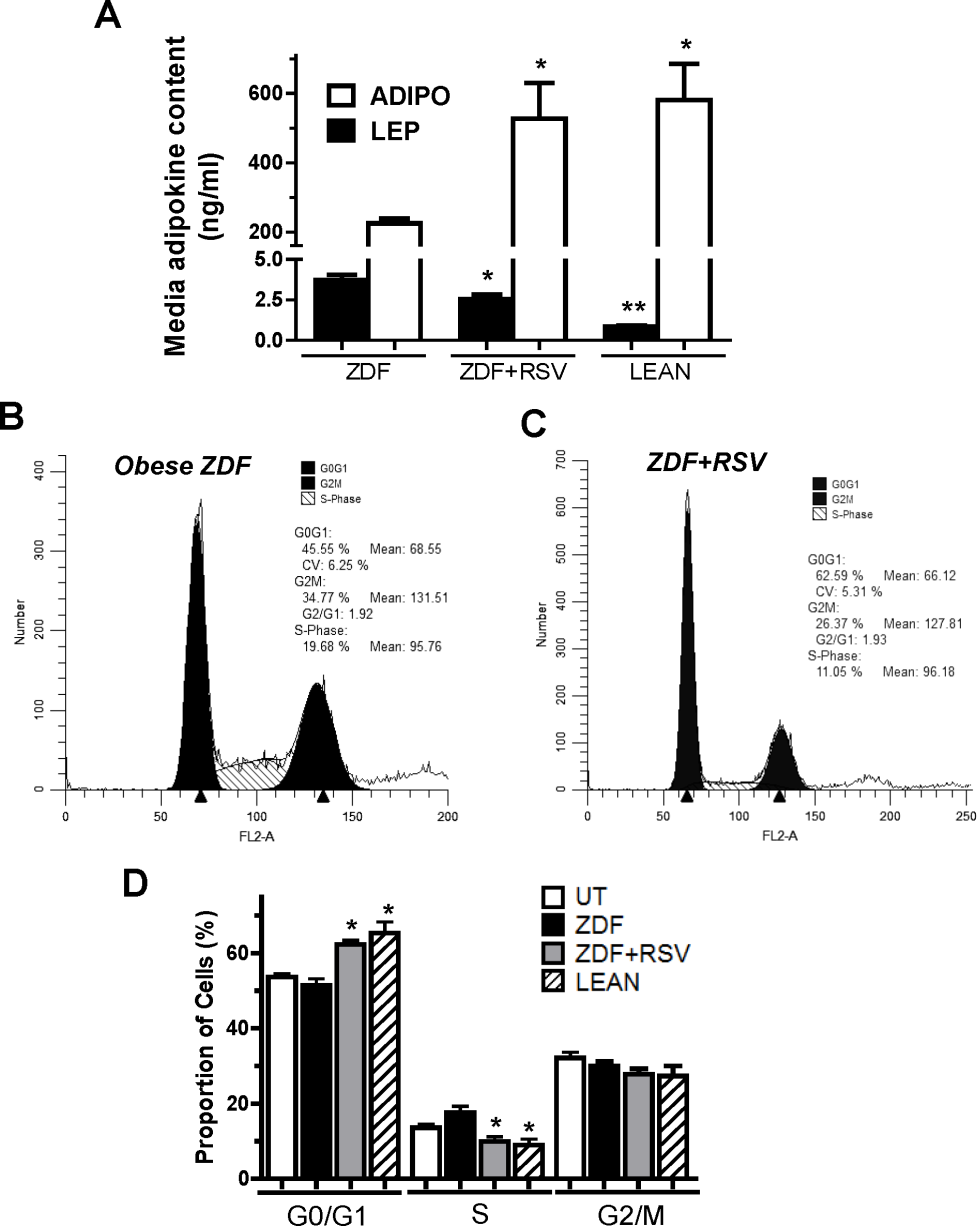
ZDF+RSV-CM supplementation causes cell cycle exit in MCF7 cells compared to ZDF-CM. A: Graphical representation of ADIPO and LEP concentration (ng/ml) in conditioned media (CM) created from scAT. B-C: Typical cell cycle profiles in MCF7 cells grown in CM prepared from scAT of ZDF or ZDF+RSV supplemented rats. D: Graphical representation of multiple cell cycle profile experiments observing effects of ZDF-CM (closed bars), ZDF+RSV-CM (grey bars) and lean Zucker (hatched bars) in proliferating MCF7 cells. * denotes significantly different from ZDF (p<0.05) as determined by Tukey’s post-hoc tests (p<0.05, n = 5-6/group).

After determining that isolated adipocytes alone can alter the tumor growth environment with isolated ZDF adipocytes inducing cell cycle entry in arrested MCF7 cells, we set out to determine whether these effects were similar to those of whole adipose tissue. Additionally, we wanted to establish that RSV supplementation can alter the effects of obese ZDF adipose tissue secretion profile, which would elicit altered cell cycle effects in proliferating MCF7 cells. We used a model where crude scAT preparations were used to create a conditioned media (CM) and MCF7 cells were subsequently incubated with the CM. We employed 3 groups for these experiments (lean, ZDF and ZDF+RSV). Zucker (lean) rats were lighter than ZDF animals and RSV supplementation did not affect body mass of the ZDF animals (381±7 g vs. 383±5 g), respectively [21]. Additionally, the levels of both IL-6 (5.28±0.80 vs. 4.38±0.60 pg·ml^-1^·mg tissue^-1^) and TNF-α (261±44 vs. 400±106 μg·ml^-1^·mg tissue^-1^) produced from isolated inguinal scAT was found to be no different (p>0.05) between ZDF and ZDF+RSV groups, respectively, as previously reported [21]. This initially suggested that there was not a significantly increased inflammatory response between ZDF and ZDF+RSV animals. CM prepared from scAT of ZDF animals (ZDF-CM) contained LEP levels (3.7±0.8 ng/ml) that were 4.5-fold higher (p<0.05) than those in CM prepared from scAT of lean Zucker rats (lean-CM; 0.8±0.1 ng/ml; Fig 4A). Lean-CM contained ADIPO levels (580.7±181.7 ng/ml) that were 2.6-fold higher (p<0.05) than those in ZDF-CM (225.1±32.5 ng/ml; Fig 4A). RSV supplementation in ZDF animals appeared to elicit alterations in the adipokine profile secreted by scAT into CM (ZDF+RSV-CM). RSV supplementation caused a 2.3-fold increase (p<0.05) in ADIPO (527.3±130.7 ng/ml) compared to ZDF-CM (Fig 4A). Furthermore, ZDF+RSV-CM demonstrated a 32% lower level of LEP (2.6±0.8 ng/ml) compared to ZDF-CM (p<0.05; Fig 4A). These observed changes resulted in vast differences in the overall stoichiometric ADIPO:LEP ratios among groups. ZDF-CM contained the lowest ADIPO:LEP ratio (61:1), while lean-CM had the highest ADIPO:LEP ratio (696:1). RSV altered the adipokine secretion profile such that it had an ADIPO:LEP ratio that was 3.4-fold higher (207:1) than ZDF-CM (p<0.05).

Given our intent to determine whether RSV supplementation altered the tumor growth environment created by adipose tissue, we measured the cell cycle profiles of MCF7 cells grown in the CM using FACS analyses (Fig 4B and 4C). ZDF+RSV-CM induced an increase (p<0.05) in the proportion of MCF7 cells in G0/G1 by 24% (62.5±2.3% vs. 50.5±2.8%; Fig 4D) compared to ZDF-CM. ZDF+RSV-CM concomitantly reduced (p<0.05) the number of cells in S-phase compared to ZDF-CM treated MCF7 cells by 47% (9.8±3.0% vs. 18.5±2.1%; Fig 4D), respectively. Despite not inducing any changes in body weight, RSV supplementation altered the endocrine function of the adipose in ZDF animals such that it elicited similar effects as lean-CM on MCF7 cell cycle regulation.

### RSV-dependent alterations in scAT adipokine secretion profile MCF7 protein expression

**Fig 5:**
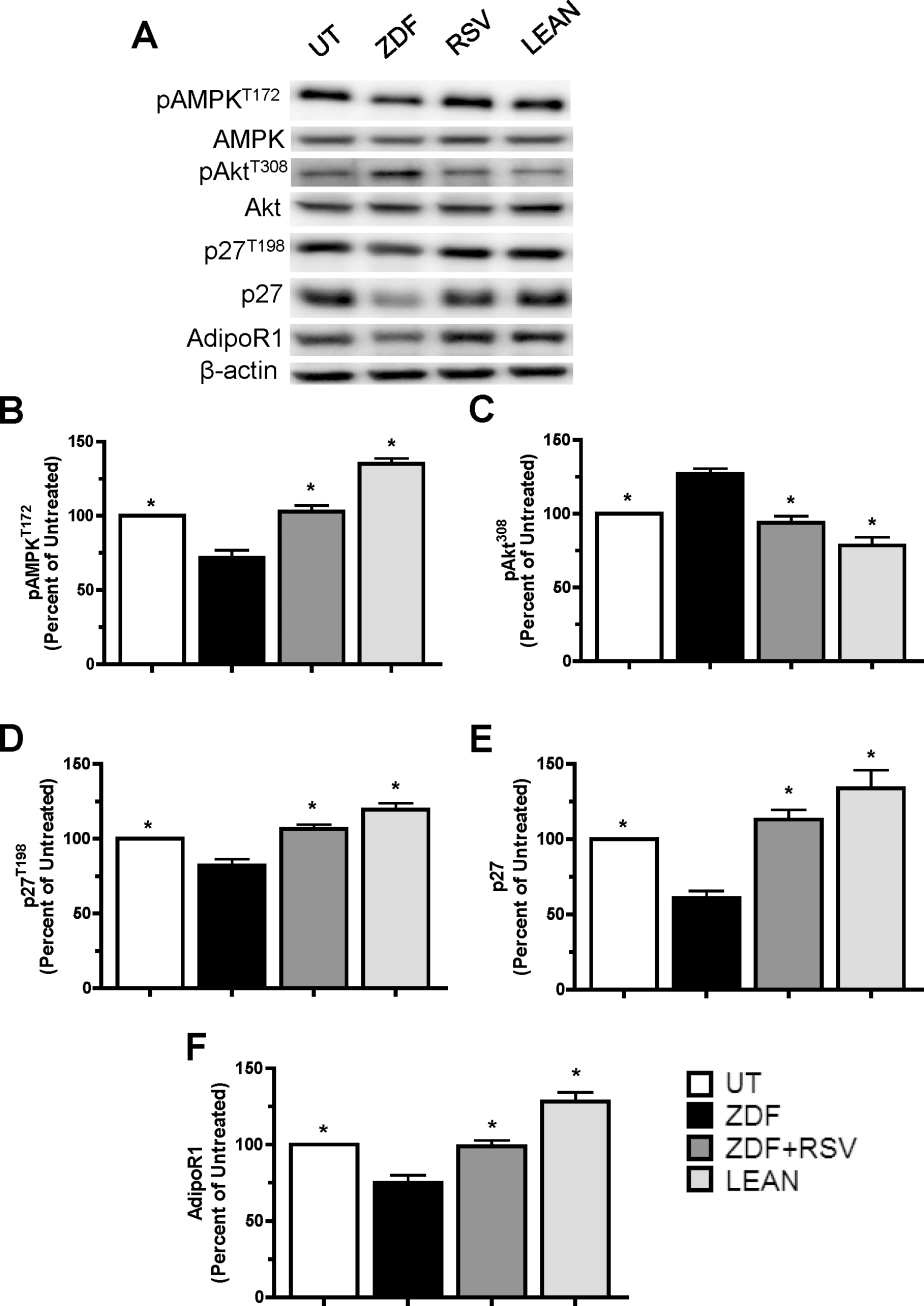
ZDF rats supplemented with RSV prevents the deleterious effects of ZDF-CM on cell cycle proteins. A: Representative western blots for selected proteins showing the effects of treatment with CM prepared from UT (open bar), ZDF-CM (closed bar), ZDF+RSV-CM (grey bar) and lean Zucker-CM (hatched bar) on proliferating MCF7 cells. B-F: Graphical representations of multiple experiments showing the effects of CM on pAMPK^T172^, pAKT^T308^, p27, p27^T198^ and AdipoR1 protein levels. β-actin was used as a loading control. One-way ANOVAs showed significant main effects for all experiments (p<0.05). * denotes significantly different from ZDF (p<0.05) as determined by Tukey’s post-hoc tests; ** indicates significantly different from all other groups (p<0.05; n = 5-6/group).

Given the effects RSV supplementation elicited on adipose regulation of MCF7 cell cycle growth, we evaluated intracellular signaling and cell cycle proteins to unravel the molecular mechanisms underlying these changes. As found using our co-culture model, ZDF-CM was found to decrease pAMPK^T172^, p27, p27^T198^ and AdipoR1 in MCF7 cells compared to those cells grown in cells growing lean-CM by 47%, 51%, 31% and 37%, respectively (p<0.05). In addition, ZDF-CM increased MCF7 cell pAkt^T308^ by 39% compared to cells grown in lean-CM (Fig 5A–5F). Compared to ZDF-CM, cells grown in ZDF+RSV-CM showed increased levels of pAMPK^T172^ (43%), p27 (85%), p27^T198^ (30%) and AdipoR1 (22%), while exhibiting decreased levels of pAkt^T308^ (34%) proteins (Fig 5A–5F; p<0.05). Thus, RSV supplementation of obese animals altered the growth microenvironment created by scAT to a point that it elicited identical anti-proliferative and protein expression effects on MCF7 cell growth as did scAT from lean animals.

## Discussion

Adipose tissue is increasingly being highlighted as an important endocrine tissue. The effects of adipose-derived hormones on numerous tissue types are being continually described in the literature. Included in the tissue types affected by adipocytokines are numerous different cancer subtypes. ADIPO and LEP are the two most abundant adipokines and as such are among the most intensively examined and have each been shown to affect the growth status of breast cancer cells [26], making them prime candidates responsible for the molecular link between obesity and breast cancer. Obese breast cancer patients exhibit lower ADIPO:LEP ratios compared with lean patients, which associates with advanced tumors and poorer patient prognosis [27,28]. It is known that ADIPO and LEP activate AMPK [7,29] and Akt [8] signaling pathways, respectively, and these pathways antagonize each other in breast cancer cells (28). Our data support this, as scAT from “obese” ZDF rats elicited decreases in pAMPK^T172^ and increases in pAkt^T308^ in MCF7 cells compared to scAT from lean animals in both co-culture (Fig 3) and CM experiments (Fig 5). These observed effects of adipose on MCF7 growth persist whether the cells are proliferating or in a quiescent state. The downstream cell cycle effects of this AMPK/AKT antagonism was manifested by decreases in the levels of p27, p27^T198^ and AdipoR1 and increases in cyclin E, indicating increased proliferation, which was confirmed using FACS analyses. Thus, it appears in an obese vs lean adipose-dependent tumor growth microenvironment that the decision whether to enter or exit the cell cycle lies in the relative effects that the adipose tissue exerts on these two pathways via their respective receptors on the cell surface. Although we identify ADIPO and LEP as important likely regulators of this external milieu, the possibility exists that a multitude of the more than 400 adipokines secreted by adipose tissue are also involved. Furthermore, there may be contributions from inflammatory cytokines that are associated with obesity development, although we did not measure any significant differences in the present study between ZDF and lean animals. Regardless, it seems that ADIPO and LEP can serve as reliable predictors of the overall tumor growth microenvironment in any individual, regardless of adiposity, making them potential clinical markers of the tumor growth environment [15]. Interestingly, RSV imparted beneficial effects on adipose-dependent tumor growth microenvironment without eliciting changes in body weight [21]. Thus, it is the phenotype of the adipose rather than the absolute amount that drives the adipose-dependent contribution to the growth environment, and inducing differences in adipokine secretion profile would seem to be a more prudent measure of intervention success than actual changes in typical anthropometric measures of adiposity.

Adipose tissue is comprised of many cell types including adipocytes, fibroblasts, macrophages, stromal cells and endothelial cells. Since each of these cells are capable of producing and secreting growth factors [30], determining the specific component of the adipose tissue responsible for these adipose-dependent effects in lean and obese patients is difficult. Much research suggests that the inflammatory response that is associated with obesity is responsible for the observed endocrine/paracrine cancer effects associated with obesity. However, we were unable to detect statistically significant differences in inflammatory cytokines that are traditionally associated with obesity including TNF-α and IL-6, two standard markers of an inflammatory response [31]. Thus, it appears that despite established anti-inflammatory effects of RSV, we observed no reduction in the inflammatory markers secreted from the adipose of RSV treated ZDF animals [21]. The lack of difference in the inflammatory activity of adipose from ZDF and ZDF+RSV animals may be due to the lack of weight loss observed in RSV supplemented animals. Given the strong association between adiposity and inflammation there may be no expected difference in the inflammatory background between groups in the absence of differences in body mass. As such, this does point to any differences brought about by RSV treatment being due to the adipocyte portion of the adipose tissue. This is in agreement with our initial experiments (Figs 1–3) where purified adipocytes from ZDF animals induced S-phase entry in arrested MCF7 cells while adipocytes from lean controls promoted further cell cycle withdrawal, all in the absence of any inflammatory cells. However, while there is evidence that the adipocyte component plays a highly important role in mediating the creation of an obesity-dependent deleterious tumor growth microenvironment, these results do not explicitly rule out a role for inflammatory cells and important inflammatory cytokines (i.e. TNF-α) to established obesity-dependent effects [32]. When examining paracrine vs. endocrine effects questions often arise around the adipose depot being studied. These are valid concerns as different cancers will share proximity to different adipose tissue depots. Since visceral and subcutaneous adipose depots can respond differently to the same stimulus, our use of scAT has a relevance to breast cancer. However, the induction of adiposity via high-fat diet elicits the exact same responses in visceral depots that we report here using scAT [15], suggesting an overall homogeneity of response between the two adipose depots with respect to their ability to contribute to the overall tumor growth microenvironment. Furthermore, we conducted our co-culture experiments using purified visceral adipocytes and found identical responses using lower total adipocyte volumes, which is consistent with published results suggesting that visceral adipose is more active than scAT (Connor and Sauvé, unpublished).

RSV directly inhibits breast cancer cell growth in cell culture, but these effects are controversial [33–35]. However, here we have identified important secondary effects of RSV with relevance to breast cancer cells, mediated by altering the adipokine secretion profile of adipose tissue *in-vivo* (Figs 4 and 5). RSV supplementation altered adipocyte function such that ZDF+RSV-CM abolished the effects elicited on intracellular protein expression in MCF7 cells by ZDF-CM, resulting in decreased cell proliferation (Fig 4D). RSV increased circulating ADIPO via the production and secretion of ADIPO from scAT [21], and decreased the production of LEP. Given the antagonism between ADIPO and LEP signaling pathways we evaluated the ADIPO:LEP ratio as an indicator of the tumor growth microenvironment. RSV ameliorated the effects of obesity in a similar manner as low volume physical activity has been previously shown to [15]. However, RSV elicited greater changes in ADIPO than LEP secretion, while exercise caused greater changes in LEP than ADIPO secretion. Thus, despite differences in the specific changes in each adipokine, the ADIPO:LEP ratio was altered in a similar fashion and the overall effect of these two obesity-targeted interventions on MCF7 cell proliferation was identical. Examination of only one of these adipokines would not be completely indicative of the total efficacy of each intervention and could result in incomplete interpretations of the effectiveness of both RSV and exercise. Given the antagonistic intracellular effects of ADIPO and LEP in combination with the fact that a cancer cell will be exposed to both adipokines *in vivo*, the case for using the ADIPO:LEP ratio as a more accurate predictor of tumor growth microenvironment is supported by our results.

The current study isolated the effects of adipose/adipocytes *ex vivo* on MCF7 cell regulation growth. MCF7 cells are a long established model for ER-positive breast cancer. Furthermore, the intracellular signaling pathways and mediators in this cell line remain intact, allowing for the determination of which pathways are activated/deactivated in our experiments. However, they are a single cell line and not representative of all cancers thus, caution needs to be taken when interpreting the results. More work needs to be conducted on other cell lines to get a more expansive view of the efficacy of RSV in this process by evaluating these effects in cells that are ER/PR-negative, Her-2 negative and p53 negative, either individually or in any combination. It should be noted that the results herein do not appear to involve the ER/PR, the Her-2 receptor or the p53 pathway, which bodes well for future investigations and a wider ranging applicability. We were unable to show anti-inflammatory effects of RSV which have been established in the literature. It may be that a longer RSV protocol is necessary to overcome the inflammation that is associated with the severe obesity that occurs in ZDF animals. Nonetheless, this lack of anti-inflammatory effect did allow for an evaluation of the contribution of the adipocyte portion of the adipose tissue as the effects occurred despite similar inflammatory conditions in the ZDF and ZDF+RSV animals. However, further work needs to be devised in order to rule out a role for inflammatory cytokines in the RSV-dependent response.

Targeting the stable components of a cancer patient’s physiology (i.e. adipose tissue) as part of cancer therapy provides a strategy that will respond reliably and predictably over time, among patients and across cancer types. The long standing relationship between obesity and cancer led us to evaluate the role of adipose tissue, and its associated adipokines, in contributing to the growth environment that any tumor is exposed to. This is because adipokine secretion profile is altered with obesity and numerous interventions including diet, exercise and nutritional supplements known to alter adipose function. RSV increases ADIPO:LEP secretion which alters the tumor growth microenvironment such that it supports cell cycle exit of the cancer cells. The current work identifies RSV as a potential supplement to augment cancer treatment, making dietary and nutritional supplementation part of a multi-faceted approach to targeting the adipose-dependent tumor growth microenvironment.
